# *PROS1* (Cys228Tyr) missense mutation associated with mesenteric and pulmonary venous thromboembolism during the COVID-19 pandemic: a case report

**DOI:** 10.3389/fcvm.2025.1610580

**Published:** 2025-07-23

**Authors:** Jingyi Huang, Yunjian Zhang, Haixu Yu, Wei Liu

**Affiliations:** ^1^ Department of Cardiology, Beijing Jishuitan Hospital, Capital Medical University, Beijing, China; ^2^ Peking University Health Science Center, Beijing, China; ^3^ Department of Pulmonary and Critical Care Medicine, Beijing Jishuitan Hospital, Capital Medical University, Beijing, China

**Keywords:** venous thromboembolism, *PROS1*, protein S deficiency, missense mutation, COVID-19, case-report

## Abstract

**Background:**

Venous thromboembolism (VTE) is influenced by both genetic and acquired risk factors, with protein S (PS) deficiency recognized as a well-established inherited thrombophilia. Introduction: We report the case of a 32-year-old male patient presenting with mesenteric venous thrombosis and pulmonary embolism caused by a missense mutation in *PROS1* during the COVID-19 pandemic.

**Methods:**

The patient presented with pleuritic chest pain and low-grade fever 15 days after a confirmed COVID-19 infection. Despite initial treatment with glucocorticoids and a macrolide antibiotic, his symptoms worsened and his D-dimer level increased. CT pulmonary angiography confirmed an acute pulmonary embolism.

**Results:**

Clinical history revealed a prior episode of mesenteric vein thrombosis and multiple acquired risk factors, including obesity, sedentariness, COVID-19 infection, glucocorticoid treatment, inflammatory response (elevated CRP and serum ferritin levels), and metabolic abnormalities (non-alcoholic fatty liver disease, hyperuricemia, and hyperlipidemia). Laboratory testing showed decreased PS activity, and genetic sequencing identified a heterozygous missense mutation in *PROS1*, c.683G>A (p.Cys228Tyr). The patient was treated with low-molecular-weight heparin (LMWH) followed by rivaroxaban. Discussion: No recurrence of VTE of bleeding events was observed during a one-year follow-up, suggesting effective management of thrombosis in the context of both inherited and acquired prothrombotic conditions.

## Introduction

Thrombophilia is an inherited or acquired medical condition characterized by an increased tendency towards abnormal blood clotting or thrombosis (1). Inherited thrombophilia is caused by various genetic mutations, including deficiencies in the natural anticoagulant proteins antithrombin, protein C, and protein S (PS), as well as the factor V Leiden and prothrombin gene mutation G20210Ac. Common acquired factors—such as advanced age, obesity, pregnancy, acute infection, inflammation, surgery, trauma, sedentariness, immobilization, and hormone-based contraceptives—may combine with inherited factors to increase the overall risk of thrombosis (2). As a vitamin K-dependent single-stranded glycoprotein, PS plays a key role in the anticoagulation pathway, primarily by serving as a cofactor for activated protein C to promote inactivation of factor Va and factor VIIIa (3). PS deficiency is an autosomal dominant trait, with an estimated prevalence of 0.03%–0.13% in the general Caucasian population (4). Patients with PS deficiency are at increased risk for venous thromboembolism (VTE), including deep vein thrombosis or pulmonary embolism (5). We report the case of a 32-year-old male patient who presented with mesenteric venous thrombosis and pulmonary embolism caused by a heterozygous missense mutation in *PROS1*, c.683G>A (p.Cys228Tyr) during the COVID-19 pandemic.

## Case presentation

**Figure 1:**
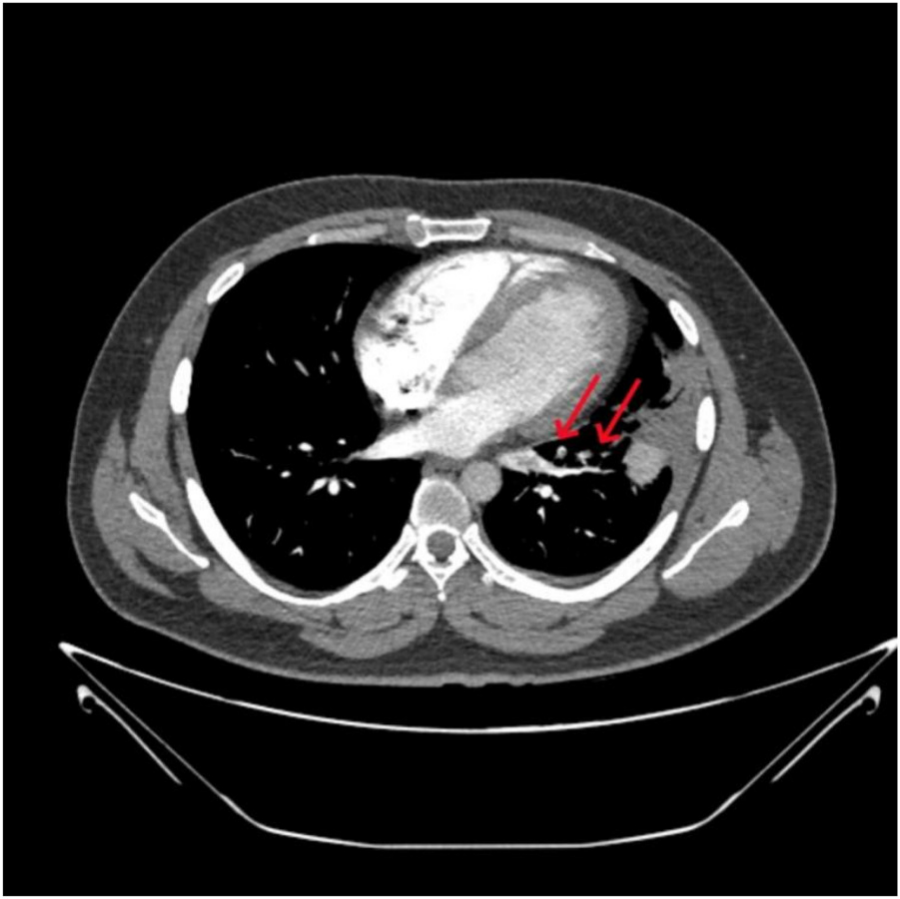
CTPA demonstrated confirmed pulmonary embolism before admission.

A 32-year-old male patient was admitted to our hospital with a 15-day history of pleuritic chest pain and low-grade fever, both of which worsened with exertion and deep breathing. Initial chest CT imaging raised suspicion of an infection in the left upper lobe, and empirical treatment was initiated with quinolone antibiotics and methylprednisolone. However, over the following 10 days, the patient's chest pain and wheezing progressively worsened. On presentation, the patient had stable vital signs, no signs of hemodynamic instability, an oxygen saturation of 97% on room air, and a simplified Pulmonary Embolism Severity Index (sPESI) score of 0. CT pulmonary angiography (CTPA) revealed a pulmonary embolism in the lingular segment of the left upper lobe and the anteromedial basal segment of the left lower lobe (Figure 1). D-dimer was elevated at 5.09 mg/L FEU, although no thrombosis was detected in the lower extremity veins. Coagulation tests on admission revealed thrombin time of 21.8 s↑, fibrin degradation products of 735 mg/dl↑, and D-dimer of 5.09 mg/L FEU↑. Thrombophilia screening showed decreased PS activity (53.3%↓), while antithrombin III and protein C activities were within normal limits. Tests for lupus anticoagulant, anti-cardiolipin antibody, anti-phosphatidylserine/prothrombin antibodies, anti-nuclear antibodies, and anti-neutrophil cytoplasmic antibodies were all negative. Liver and lipid panels showed elevated alanine aminotransferase (82 IU/L↑), aspartate aminotransferase (68 IU/L↑), and gamma-glutamyl transpeptidase (97 IU/L↑). Total cholesterol and triglyceride levels were 5.69 mmol/L↑ and 2.21 mmol/L↑, respectively. High-sensitivity C-reactive protein was elevated at 17.65 mg/L. Fungal and bacterial infection markers, including 1,3-β-D-glucan test (G test), galactomannan test (GM test), and procalcitonin test (PCT) were all negative, ruling out invasive infections.

The patient's medical history included a three-year history of nonalcoholic fatty liver disease, a prior episode of mesenteric vein thrombosis one year earlier, and a confirmed COVID-19 infection within the preceding month. There was no known family history of VTE. The patient was overweight (BMI 29.4 kg/m²) and regularly smoked and consumed alcohol.

**Figure 2:**
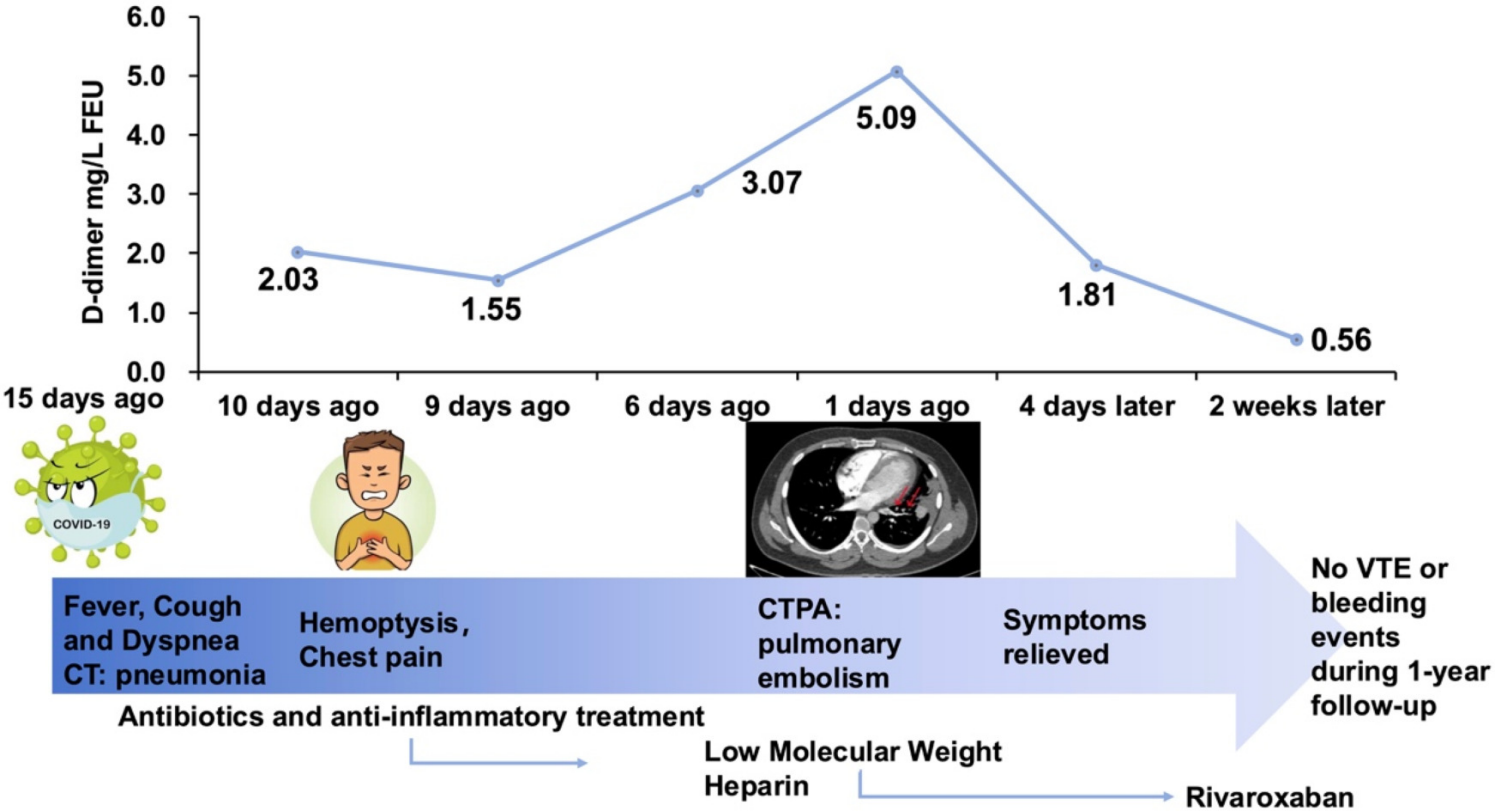
Summary timeline of symptoms, interventions, key diagnostics and outcomes. FEU, fibrinogen equivalent unit; CT, computed tomography; CTPA, computer tomography pulmonary angiography; VTE, venous thromboembolism.

The patient was diagnosed with acute pulmonary embolism (low-risk, according to the ESC guidelines) and started on low-molecular-weight heparin (LMWH) 8,000 IU every 12 h. Given the concurrent diagnosis of community-acquired pneumonia, cefoperazone-sulbactam was administered alongside hepatoprotective agents. Both the patient's symptoms and D-dimer levels significantly improved (Figure 2). LMWH was subsequently transitioned to rivaroxaban 15 mg twice daily for 3 weeks, followed by 20 mg once daily. Following the standard anticoagulation therapy, rivaroxaban was continued at a reduced dose of 10 mg once daily for extended preventive anticoagulation (6). No venous thromboembolism or bleeding events were reported during the 1-year follow-up period.

**Figure 3:**
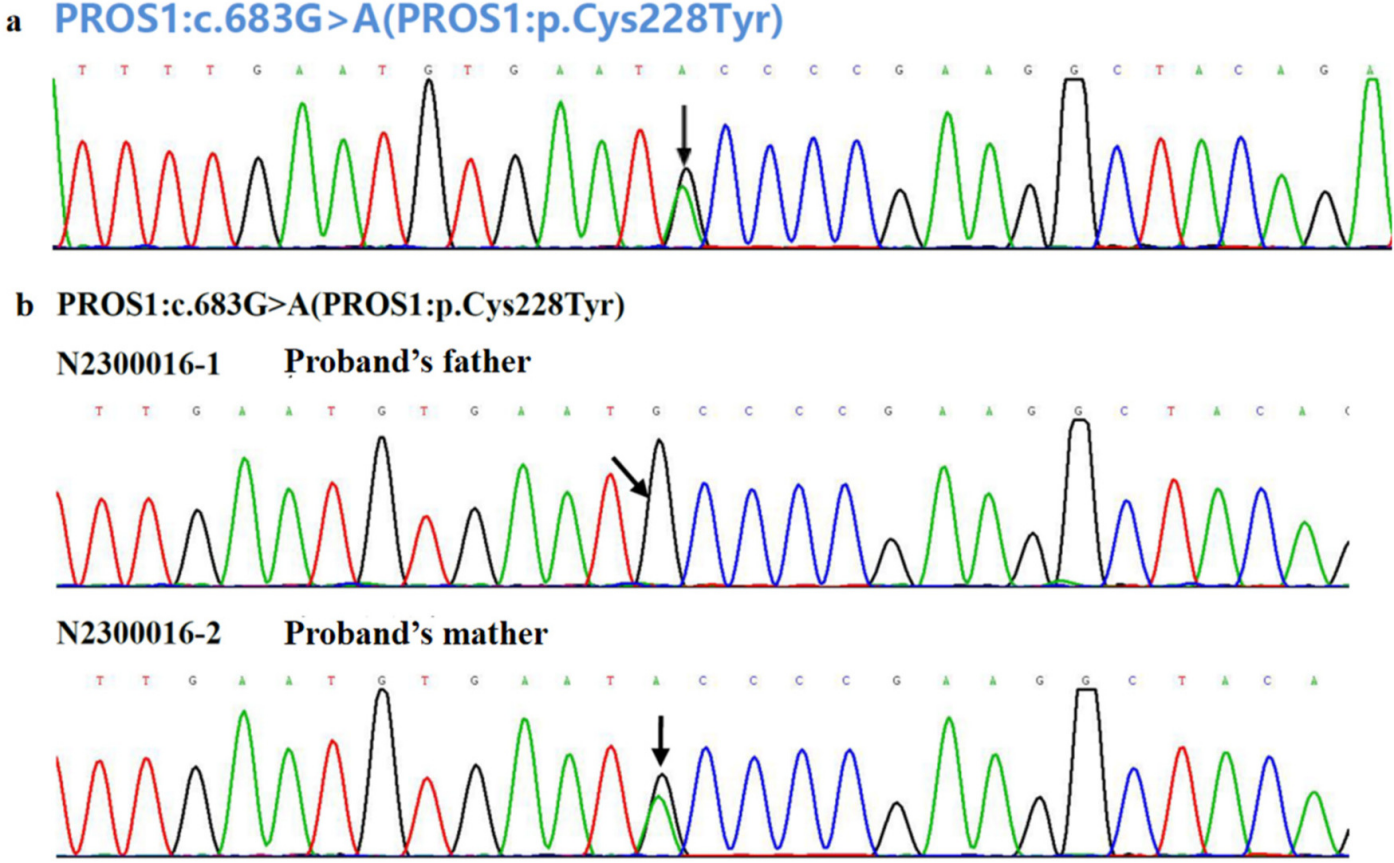
**(a)** Genetic analysis for the causal mutation of the patient with VTE and **(b)** his parents. The black arrow indicates the base pair substitution site.

**Figure 4:**
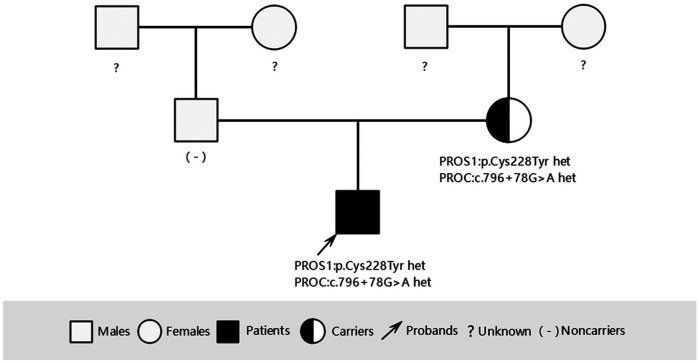
Three-generation pedigree of the patient with VTE.

To investigate the cause of the thromboembolism, thrombophilia screening was performed. PS activity was markedly reduced (53.3%; male reference range: 63.5%–149%). Genetic analysis was conducted to identify potential genetic causes of PS deficiency. A combination of single-gene Sanger sequencing and next-generation sequencing (NGS), including whole-exome sequencing (WES), was employed, achieving 99.93% genome coverage with an average coverage depth of 112×. Genes encoding anticoagulant proteins, coagulation factors, and fibrinolytic regulators were analyzed. A rare heterozygous mutation in the *PROS1* gene (c.683G>A; p.Cys228Tyr) was identified (Figure 3a). This variant was present in both the patient and his mother but absent in his father (Figures 3b, 4).

### Patient perspective

The patient's symptoms improved following diagnosis, and an appropriate therapeutic plan was established. During hospitalization, the patient expressed comfort and reassurance after engaging in shared decision-making with the medical team. He also acknowledged the valuable support provided by both the healthcare provider and family members, which contributed to his rehabilitation and clinical outcomes during the 1-year follow-up period.

## Discussion and conclusion

In this study, we report the case of a young, overweight male with multiple acquired thrombotic risk factors, including pneumonia, COVID-19 infection, glucocorticoid use, inflammatory activation, and metabolic disorders, who presented with mesenteric venous thrombosis and recurrent VTE. Considering the reduced PS activity and normal findings in other thrombophilia-related tests (including lupus anticoagulants, antiphospholipid antibodies, and tumor biomarkers), genetic screening was performed for inherited thrombophilia. A heterozygous missense mutation in *PROS1* (c.683G>A, p.Cys228Tyr) was identified, which has not been reported previously. The patient was treated with enoxaparin, followed by rivaroxaban, and experienced no adverse events during a 1-year follow-up.

PS is an anticoagulant and its deficiency increases the risk of VTE. PS deficiency occurs in approximately 2% of patients with unselected VTE and in 1%–13% of patients overall (7). In a Han Chinese population-based case-control study, 8.5% (51/603) of patients with VTE exhibited PS activity deficiency (8). Acquired PS deficiency may result from warfarin use, pregnancy, hepatic dysfunction, nephrotic syndrome, chronic infections (e.g., HIV), or disseminated intravascular coagulation (9). Hereditary PS deficiency—an autosomal dominant disorder—results from mutations in the *PROS1* gene (located at 3q11.2), which comprises 15 exons and spans over 80 kb of genomic DNA. More than 200 mutations in *PROS1* gene have been reported in patients with PS deficiency, approximately 50% of which are point mutations (10). Missense mutations primarily cause quantitative PS deficiency through defective synthesis, stability, or secretion of mutated proteins. In this case, we identified a missense mutation in PROS1 (c.683G>A, p.Cys228Tyr), which likely represents a causal variant for the loss of PS function and contributes to an increased risk of VTEs, such as pulmonary embolism and mesenteric venous thrombosis. Most patients with hereditary thrombophilia experience their first thrombotic episode before the age of 45 (7), consistent with this case. Additionally, the patient's pulmonary embolism likely resulted from recurrent venous thrombosis, a recognized feature in patients with antithrombin, protein C, or PS deficiency (11). The patient harbored a heterozygous missense variant in the *PROS1* gene: c.683G>A (p.Cys228Tyr). This variant was not identified in major population databases, including the 1,000 Genomes Project, ESP6500, and ExAC, and was also absent in both thrombotic cases and controls within the BGI local cohort. In-silico pathogenicity prediction tools uniformly indicated damaging effects: SIFT “D,” PolyPhen-2 “D,” MutationTaster_pred “D,” VEST4 score 0.960, REVEL score 0.959, with aggregate predictions of “5D/1H”. Regarding the biochemical impact and conservative analysis, the mutation results in the substitution of cysteine with tyrosine—both polar, uncharged amino acids—at a site highly conserved across vertebrate orthologs. This supports the likelihood of deleterious structural or functional consequences. The variant was not identified in ClinVar and Human Gene Mutation Database (HGMD) databases. However, neighboring residues are also implicated in PS deficiency; c.701A>G (p.Tyr234Cys) is a known pathogenic variant associated with autosomal recessive PS deficiency, while both c.682T>A (p.Cys228Ser) and c.684C>G (p.Cys228Trp) are classified as pathogenic or likely pathogenic in HGMD. Considering the variant's extreme rarity, consistent in-silico deleterious predictions of pathogenicity, strong evolutionary conservation, and proximity to established pathogenic variants, c.683G>A (p.Cys228Tyr) is classified as a likely pathogenic (Class B) mutation, according to the current guidelines. Nonetheless, the absence of functional assays and segregation data precludes a definitive pathogenic classification.

Interestingly, although the same *PROS1* mutation was identified in the patient's mother, she had no documented history of venous thrombosis, suggesting that additional environmental or physiological triggers were necessary for thrombus formation. In addition to well-known risk factors such as inflammation, surgery, and trauma, COVID-19 might have acted as a triggering event for this patient. Notably, COVID-19 enhances thrombin production and increases the von Willebrand factor and factor V levels, resulting in a hypercoagulable state (12). Additional modifiable lifestyle-related risk factors for VTE include obesity, sedentariness, and immobilization. Therefore, both the patient and his mother were educated to avoid potential thrombotic risk factors.

Anticoagulation is considered the primary therapy for acute pulmonary embolism. Anticoagulants include parenteral anticoagulation, vitamin K antagonists (VKA), and direct oral anticoagulants (DOACs) (13). While conventional treatment of VTE is effective and relatively safe, it poses several limitations, including the requirement for parenteral administration of heparin and frequent monitoring of VKA (14). DOACs, which have shown comparable efficacy and improved safety profiles relative to VKA for pulmonary embolism (15) and VTE (16), offer several advantages over conventional therapy. These include fixed dosing with predictable pharmacokinetics, no requirement for laboratory monitoring, and minimal food and drug interactions, all of which may improve medication adherence (17, 18). A prospective cohort study demonstrated DOACs' association with lower 2-year VTE recurrence post-discontinuation compared to heparin/VKA in patients with inherited thrombophilia and VTE. Additionally, this study showed significantly higher overall bleeding rates with DOACs than with heparin/VKA (6). In this case, the patient received continued rivaroxaban treatment after the initial therapy, as the patient's clinical profile suggested a low bleeding tendency and the benefits from extended anticoagulation outweighed the risk of bleeding.

In short, inherited PS deficiency due to a heterozygous missense mutation in *PROS1* (p.Cys228Tyr) represented the genetic basis in this patient, and long-term anticoagulant therapy was beneficial.

## Data Availability

The original contributions presented in the study are included in the article/Supplementary Material, further inquiries can be directed to the corresponding authors.
